# A rare cause of gastrointestinal bleeding in adults: Ileal duplication with ulceration: A case report and review of literature

**DOI:** 10.3389/fsurg.2022.927288

**Published:** 2022-08-12

**Authors:** Lingli Li, Hongping Li, Xiaoyan Chen, Jin Yao, Rui Xie, Hong Wang

**Affiliations:** ^1^ Department of Gastroenterology, Digestive Disease Hospital, Affiliated Hospital of Zunyi Medical University, Zunyi, China; ^2^ Department of Pathology, Affiliated Hospital of Zunyi Medical University, Zunyi, China

**Keywords:** alimentary tract duplication, ileal duplication, ulcer, gastrointestinal bleeding, double-balloon enteroscopy (DBE), surgery

## Abstract

The ileal duplication, which is a congenital anatomical abnormality of the digestive tract, can lead to the manifestation of the alimentary tract duplication in the small intestine. It is also the rare cause of gastrointestinal bleeding and usually seen in infants, but only rarely in adults. Herein, we describe a case of adult male was suffering from intermittent massive hematochezia for three years, accompanied by abdominal pain, syncope, and palpitations. However, no positive findings were found by gastroscopy and colonoscopy in other hospitals. He visited our hospital owing to the recurrent hematochezia, but re-examination by gastroscopy and colonoscopy indicated no significant abnormality, and hence small intestinal vascular malformation bleeding was considered. Therefore, double-balloon enteroscopy (DBE) examination was conducted and the results revealed a double-lumen opening in the ileum, which was 100 cm from the ileocecal valve. The blind end was observed 6 cm away from one opening along the depth direction, and an ulcer was observed on the intestinal wall of the blind segment, which was considered as an ileal duplication. The patient thereafter underwent surgery subsequently. Postoperative pathology confirmed ileal duplication and that gastrointestinal bleeding was primarily caused by ulcer hemorrhage. The patient had no discomfort after the follow-up. In this report, we have reviewed and summarized the literature to provide references for both diagnosis and treatment of ileal duplication.

## Introduction

Initially proposed in 1937 (1), alimentary tract duplication constitutes a rare group of congenital developmental disorders that refers to the appearance of a tubular or cyst cavity-like structure adjacent to the digestive tract that shares various features with the primary digestive tract. It can present itself singly or multiply anywhere in a digestive tract. Moreover, its incidence rate has been variably reported and it primarily occurs in the pediatric period but rarely observed in adults (2). The disease can exhibit diverse clinical manifestations, and patients might exhibit gastrointestinal bleeding, abdominal pain and intestinal obstruction, even asymptomatic; the specific etiology remains unclear. The ileal duplication is the most common (about 30%) condition among the various alimentary tract duplications (3). It is typically observed in the terminal ileum and is diagnosed postoperatively. Interestingly, with the rapid development of endoscopic technology in recent years, double-balloon enteroscopy (DBE) has been demonstrated to be an effective way to diagnose ileal duplication in adults (4). This article reports a 45-year-old male with unexplained gastrointestinal bleeding who was diagnosed with ileal duplication with ulcer by DBE followed by confirmation by postoperative pathology, with the aim of providing ideas for the diagnosis and treatment of ileal duplication.

## Case presentation

A 45-year-old man was admitted to the emergency department of our hospital on 03 Nov 2021 due to intermittently blood-stained stools for three years and recurrence over the past three days. It was found that during the past three years, the patient experienced the fresh blood-stained stool discharge with no obvious cause twice, but the quantities were uncertain each time. This condition was also accompanied by periumbilical pain, palpitation, fatigue, and dizziness. Subsequently, he experienced a transient loss of consciousness, but did not display other symptoms such as nausea, vomiting, hematemesis, fever and abdominal distension. The patient condition was reviewed by a local hospital and no bleeding points were found upon examinations using gastroscopy and colonoscopy. Intestinal atrio-venous malformation (AVM) bleeding was considered as a possibility, but no enteroscopy examination was performed. He was discharged after the hemostatic treatment. Thereafter, the patient discharged bright red watery stools for three times three days ago (the total amount was about 500 ml), which was also accompanied by abdominal pain, dizziness, fatigue, and four episodes of syncope. Hence, he visited our hospital for further medical assessment and treatment.

**Figure 1:**
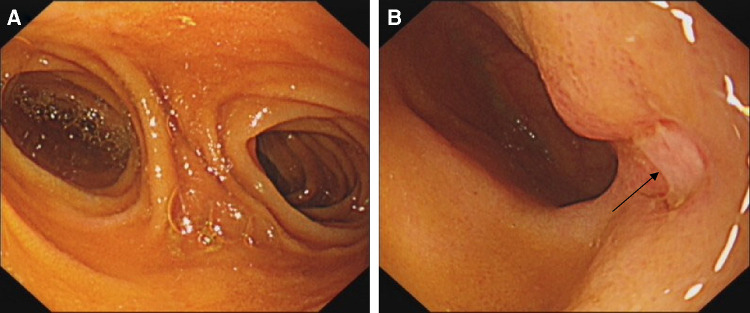
DBE: (**A**) double-lumen like opening was seen in ileum about 100 cm away from the ileocecal valve; (**B**) ulcer (black arrow) was observed on the sidewall of the duplicated intestinal canal.

**Figure 2:**
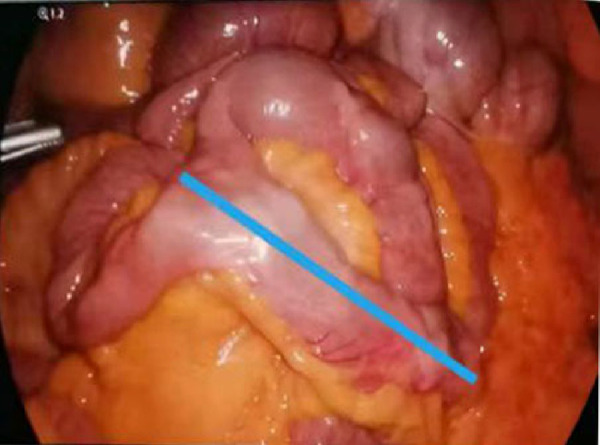
Ileal duplication (blue line) with a length of about 6 cm was observed during the laparoscopy.

**Figure 3:**
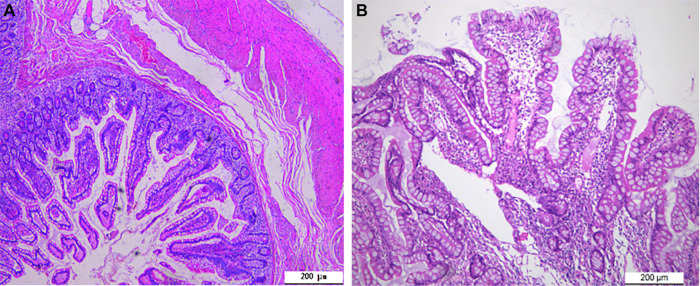
Pathological results. (**A**) The duplicated intestinal canal exhibited the intestinal wall structure of the normal ileum (H.E. × 100); (**B**) Inflammatory cell infiltration was observed at the edge of the ulcer of the duplication intestine, without the presence of heterotopic gastric mucosa (H.E. × 100).

Physical examination on admission: Heart rate was 80 beats/min and blood pressure was 106/68 mmHg. The abdomen was flat and soft with mild periumbilical tenderness, bowel sounds were normal, and the rest of the abdomen displayed no significant positive signs. Blood routine test showed that hemoglobin content was 108 g/L, and the review of gastroscopy and colonoscopy revealed no positive signs. Total abdominal Computed Tomography (CT) plain scan + enhancement displayed no significant abnormalities. Small intestinal bleeding (AVM) was first considered based on his medical history and the examination results. Hence, DBE examination was performed: The lens entered from the anus to the ileum, and a double-lumen opening was observed approximately 100 cm away from the ileum (Figure 1A), with the blind end visible 6 cm away from one of these openings. Upon slow removal of the lens, a 0.5 cm × 0.6 cm ulcer covered by white moss was observed on the lateral wall, with congested and edematous surrounding mucosa but no active bleeding was observed (Figure 1B). The tissue was obtained from the ulcer edge for further examination. The pathological report showed chronic inflammation accompanied with mild acute changes (Figure 3B). According to his medical history and enteroscopy results, ileal duplication with ulcer bleeding was regarded as the primary cause of gastroenteric bleeding. Surgery was thereafter performed. During the operation, a repeated ileum segment (about 6 cm) was found 100 cm away from the terminal ileum, with complete mesangium and the blood supply (Figure 2). The ileal duplication segment was resected followed by the pathological examination. However, under the microscope, the duplication intestinal canal exhibited the intestinal wall structure of normal ileum, but no heterotopic gastric mucosa was found (Figure 3A). The patient was diagnosed with ileal duplication, and there was no recurrence of blood-stained stool during the postoperative follow-up.

## Discussion

Clinically, intestinal duplication manifesting itself as gastrointestinal bleeding is relatively common. However, it still remains a clinical challenge to accurately identify the potential cause of gastrointestinal bleeding owing to its low overall incidence rate (especially in adults). It might also lead to the various life-threatening consequences when diagnosed in severe form. Therefore, increased awareness about this group of disorders is needed to avoid misdiagnosis and missed diagnosis that might lead to the aggravation of the patient's condition. Herein, various previously reported case reports similar to the present case have been reviewed and summarized (see Table 1).

**Table 1:** Literature review of ileal duplication reported in adults combined with gastrointestinal bleeding.

Authors	Year	Age	Gender	Bleeding site	Bleeding performance	Combination with an ulcer, perforation, or other abnormalities	Shape	Histological type	Diagnostic modality	Treatment
Ammann J et al.	1975	27	F	Distal ileum	Hematochezia	Ulcer	Unknown	Duplication with gastric mucosa	Exploratory laparotomy	Surgery
Salameh RN et al.	1984	30	M	Adjacent to the ileocecal valve	Hematochezia	Vascular malformation	Cystic	No	Gastroenterography	Surgery
Staunton DA et al.	1990	31	M	Distal ileum	Hematochezia	Ulcer	Cystic	Duplication	Radionuclide angiography; exploratory laparotomy	Surgery
Tanabe ID et al.	1995	32	M	Distal ileum	Massive Hematochezia	Ulcer	Cystic	Duplication with gastric mucosa	Visceral angiography	Surgery
Peng Y et al.	2005	19	M	30 cm from ileocecal valve	Melena	Ulcer combined with perforation	Tubular	Duplication with gastric mucosa	Exploratory laparotomy	Surgery
Yu S et al.	2005	23	M	60 cm from ileocecal valve	Melena	No	Tubular	Duplication with gastric mucosa	B-ultrasound; CT; exploratory laparotomy	Surgery
Qin G et al.	2007	25	F	40 cm from ileocecal valve	Intermittent melena	No	Tubular	Duplication	Exploratory laparotomy	Surgery
Zhao L et al.	2008	30	M	100 cm from ileocecal valve	Massive hematochezia	Ulcer	Tubular	Duplication	DBE combined with endoscopic contrast injection	Surgery
Ogino H et al	2008	35	M	1 m from ileocecal valve	Hematochezia	Diverticuloid foramen hemorrhage	Cystic	Duplication with gastric mucosa	DBE	Surgery
Wan X et al.	2009	32	M	80 cm from ileocecal valve	Hematochezia	Ulcer	Tubular	Duplication with gastric mucosa	DBE	Surgery
Wan X et al.	2009	23	F	1 m from ileocecal valve	Hematochezia	Ulcer	Tubular	Duplication with gastric mucosa	DBE	Surgery
Li W et al.	2010	28	M	The upper segment of the ileum	Hematochezia	Ulcer	Cystic	No	Intestinal double-contrast barium meal; DBE	Surgery was planned
Nadatani Y et al.	2016	73	M	100 cm from ileocecal valve	Melena	No	Tubular	No	CT; Capsule endoscopy; DBE	Surgery was planned
Takegawa Y et al.	2018	19	M	50 cm from ileocecal valve	Occult bleeding, no melena	Ulcer	Cystic	Duplication	Abdominal CT; capsule endoscopy	Surgery
Zhang et al.	2021	31	F	70 cm from ileocecal valve	Hematochezia	No	Cystic	Duplication	Abdominal CT; capsule endoscopy	Surgery

Ileal duplication usually occurs at the terminal ileum, while gastrointestinal bleeding has been primarily observed in cases where the duplication has communication with the primitive intestinal cavity. The bleeding amount is typically high, and patients present obvious melena or even massive hematochezia. Moreover, some patients have presented highly occult bleeding, no melena, and severe iron deficiency anemia due to the chronic underlying blood loss only, at which point hematological disorders need to be excluded during the diagnosis. In most cases, the specific cause of bleeding was the fact that the mucosa of the duplication of intestine contains heterotopic gastric mucosa or pancreatic tissues, which can lead to the corrosion of the intestinal wall, thus causing extensive peptic ulcer bleeding (5–7). Moreover, heterotopic gastric mucosa or pancreatic tissues can also cause acute perforation of the digestive tract (8). Nevertheless, the heterotopic gastric mucosa or pancreatic tissues present in the duplication are not enough to explain the cause of gastrointestinal bleeding in all patients with intestinal duplication. In this case study, although examination by DBE indicated an ulcer in the intestinal duplication, the postoperative pathological examination did not accurately identify such mucosal tissue, thereby indicating that ulcer formation might be attributed to various causes.

Besides gastrointestinal bleeding, patients with ileal duplication commonly suffer from abdominal pain, internal obstruction, volvulus, intussusception (9), and even malignant tumor formation has been reported in an extreme minority of patients. However, this malignant transformation typically occurs in the colonic duplication (10). For instance, Lee et al. (11) reported a case of colonic duplication combined with the papillary adenocarcinoma for the first time. Additionally, some patients can be asymptomatic for their entire life time. Therefore, the clinical manifestations of these diseases are not typical, and hence they can be often misdiagnosed or missed. Indeed, the patient reported in this case study was previously diagnosed with the small intestinal vascular malformation owing to abdominal pain and blood-stained stool. This time however, he came to our hospital and was also initially diagnosed with small intestinal vascular malformation and not correctly diagnosed until examinations by double-balloon enteroscopy and after surgery. Therefore, such disorders need to be carefully considered for patients with symptoms such as unexplained abdominal pain and gastrointestinal bleeding to avoid the potential misdiagnosis.

Ileal duplication combined with gastrointestinal bleeding is typically a critical condition and it is essential to identify the cause of bleeding in a rapid and effective diagnostic manner based on the stabilization of the vital signs and active medical hemostasis. In earlier times, however, it was not clear until an explorative laparotomy was performed due to the absence of effective examination methods to clinically analyze the small intestine. In recent years, with the rapid development of endoscopic technology, especially the application of DBE has been reported to be of significant value for the rapid diagnosis of ileal duplication (4) and can be used for hemostatic treatment under enteroscopy. Nevertheless, the use of DBE may also be limited due to the patient's own factors, such as severe intestinal stenosis or serious cardiopulmonary diseases. DBE exhibits higher specificity for the tubular type in the diagnosis of intestinal duplication but plays a minor role for the parenteral cystic type (12). A double-lumen opening or diverticulum-like orifice of the bowel might be observed during DBE examination of the tubular type (4, 13, 14). Thus, a suspected diagnosis of intestinal duplication could be made. The presentation of this case under enteroscopy was tubular ileal duplication. Besides DBE, capsule endoscopy can also be used for the diagnosis of ileum duplication, but it also carries the risk of retention (15) and thus might not achieve operations such as hemostasis. For this reason, the capsule endoscopy should be carefully considered. Other important auxiliary examinations, including B-ultrasound, CT, gastrointestinal contrast, and Magnetic Resonance Imaging (MRI), also exhibit application values in the preoperative diagnosis of intestinal duplication (16, 17). However, still the preoperative diagnosis rate is not significantly high because of the lack of characteristics. For instance, Moreillo et al. (18) recently summarized 27 different cases of isolated intestinal duplications, of which only four were diagnosed preoperatively by B-ultrasound or CT. The patient analyzed in this case study also underwent abdominal CT preoperatively, while no abnormality was identified. Currently, ^99m^Tc-pertechnetate radionuclide scanning imaging is usually employed to detect the heterotopic gastric mucosa in the duplication bowel before the operation. Indeed, ^99m^Tc-pertechnetate radionuclide scanning has been reported to have high effectiveness, with a sensitivity of up to 75% (8, 19).

Based on the previous reports, once the ileal duplication is identified, the surgical resection remains the primary treatment for the management of the disease regardless of the complications such as gastroenteric bleeding, thus achieving radical curation and good prognosis. The usual open surgery has caused significant harm to patients in the past, but now the optional laparoscope resection has significantly reduced this kind of injury and complications. The reported patient did not report any recurrent abdominal pain and blood-stained stool after laparoscopic resection of the duplication. A follow-up call with the patient indicated no major discomfort.

## Conclusion

Ileal duplication exhibits a low incidence rate in adults. It is clinically rare, easily missed as well as misdiagnosed, and can be life-threatening when identified in severe form. Hence, an increased awareness about this disorder is required. Thus, for patients with unexplained gastrointestinal bleeding observed clinically, intestinal duplication needs to be considered. We believe that DBE examination can serve as an effective preoperative auxiliary diagnostic modality, while postoperative pathology remains the “gold standard”.

## Data Availability

The original contributions presented in the study are included in the article/Supplementary Material, further inquiries can be directed to the corresponding author/s.
